# MicroRNA-34c is associated with emphysema severity and modulates SERPINE1 expression

**DOI:** 10.1186/1471-2164-15-88

**Published:** 2014-01-30

**Authors:** Santiyagu M Savarimuthu Francis, Morgan R Davidson, Maxine E Tan, Casey M Wright, Belinda E Clarke, Edwina E Duhig, Rayleen V Bowman, Nicholas K Hayward, Kwun M Fong, Ian A Yang

**Affiliations:** 1 Department of Thoracic Medicine, The Prince Charles Hospital, Brisbane, QLD 4032, Australia; 2 School of Medicine, The University of Queensland, Brisbane, QLD 4072, Australia; 3 Department of Anatomical Pathology, The Prince Charles Hospital, Brisbane, QLD 4032, Australia; 4 Queensland Institute of Medical Research, Brisbane, QLD 4006, Australia

**Keywords:** Chronic obstructive pulmonary disease, microRNA, miR-34c, Microarray

## Abstract

**Background:**

MicroRNAs (MiRNA) are small non-coding RNAs that regulate gene expression. The aim of this study was to identify miRNAs differentially expressed between mild and moderately emphysematous lung, as well as their functional target mRNAs. Resected lung from patients with COPD undergoing lung cancer surgery was profiled using miRNA (Agilent Human miRNA profiler G4470 V1.01) and mRNA (OperonV2.0) microarrays. Cells of lung origin (BEAS-2B and HFL1) were profiled using mRNA microarrays (Illumina HumanHT-12 V3) after *in vitro* manipulation.

**Results:**

COPD patients had mean (SD) age 68 (6) years, FEV_1_ 72 (17)% predicted and gas transfer (KCO) 70 (10)% predicted. Five miRNAs (*miR-34c*, *miR-34b*, *miR-149*, *miR-133a* and *miR-133b*) were significantly down-regulated in lung from patients with moderate compared to mild emphysema as defined by gas transfer (*p* < 0.01). *In vitro* upregulation of *miR-34c* in respiratory cells led to down-regulation of predicted target mRNAs, including *SERPINE1*, *MAP4K4*, *ZNF3*, *ALDOA* and *HNF4A*. The fold change in ex-vivo expression of all five predicted target genes inversely correlated with that of *miR-34c* in emphysematous lung, but this relationship was strongest for *SERPINE1* (p = 0.05).

**Conclusion:**

Differences in miRNA expression are associated with emphysema severity in COPD patients. *MiR-34c* modulates expression of its putative target gene, *SERPINE1*, *in vitro* in respiratory cell lines and *ex vivo* in emphysematous lung tissue.

## Background

MicroRNAs (miRNAs) are regulators that modulate transcription and translation of target mRNAs. There are over 1,000 miRNAs encoded within the human genome and these miRNAs are estimated to target ~60% of genes. The discovery of miRNAs has introduced a new level of complexity to be considered for every biological process. MiRNAs are implicated in normal biological functioning, including development, differentiation, homeostasis and apoptotic cell death [1]. Their dysregulation leads to disease processes including malignancy [2-5], inflammation [6,7] and heart disease [8].

MiRNAs are mediators of inflammation [9], an important mechanism driving chronic obstructive pulmonary disease (COPD) development and progression. Several miRNAs have been implicated in lung disease [10]. Exposure to cigarette smoke extract (CSE), diesel exhaust particles (DEP) and lipopolysaccharide (LPS) induce changes in lung expression of several miRNAs including *miR-26b*, *miR-31*, *Let-7a* and *miR-192*[10]. MiRNAs associated with asthma include *miR-21*, *miR-126, miR-133a*, *miR-148a/b* and *miR-152*[11]. Other miRNAs are associated with the inflammation observed in idiopathic pulmonary fibrosis (IPF) [12] and cystic fibrosis [13], and many studies implicate miRNAs in the pathogenesis of lung cancer. Collectively these studies demonstrate that miRNAs are expressed in the lung and are involved in the pathogenesis of lung disease.

COPD prevalence is continually increasing and it is predicted to become the third leading cause of death worldwide by 2020 [14,15]. COPD is under-diagnosed, since patients are most often diagnosed only when the disease is advanced, too late for prevention, and when there is no curative intervention. Better screening to detect early disease and targeted therapies to prevent progression to severe COPD are urgently needed to improve COPD outcomes. Genomic tools provide an opportunity to improve our understanding of the pathogenesis of COPD and translate this to enhanced clinical management [16]. Current understanding of COPD pathogenesis is that lung injury by inhaled tobacco results in depletion of lung alveolar tissue, large and small airway inflammation, fibrosis of small airways and mucus hypersecretion contributing to small airway obstruction. Inflammation is the main cause of lung tissue destruction and provokes defense mechanisms associated with tissue repair [17].

Given the role of miRNAs in lung inflammation and the lung’s response to inhaled toxins, miRNAs are likely to be involved in pathogenesis and/or progression of COPD. Furthermore, miRNAs regulate mRNA expression, and mRNA (transcriptome) profiles relevant to COPD pathogenesis have been identified [18-23]. MiRNA profiles in lung from patients with COPD have shown differences in miRNA expression in relation to susceptibility to COPD [24,25], when comparing moderate/severe COPD patients with non-COPD subjects [24,25]. Here we aimed to identify lung miRNAs associated with COPD severity, and demonstrate miRNA regulation of genes that may be implicated in the progression of COPD.

## Methods

### Participant selection

This study was approved by the Human Research Ethics Committees of The University of Queensland and The Prince Charles Hospital (TPCH). Lung tissue samples from 29 patients with COPD undergoing curative resection for lung cancer were selected from the TPCH tissue bank. Written informed consent was obtained from all patients prior to surgery. Detailed information on subject selection, and inclusion and exclusion criteria are listed elsewhere [18]. Briefly, smokers with at least a 20 pack year history who quit smoking at least 10 months prior to surgery, not currently on inhaled or oral steroids, and without other lung pathology that may confound spirometry measurements, were considered. All had FEV_1_/FVC (or FEV_1_/VC) ratio <0.70, indicating the presence of COPD. Twenty-nine former smokers with COPD (the ‘TPCH-KCO set’) were selected and classified by gas transfer measurements (single breath carbon monoxide diffusion coefficient, KCO) as “mild” emphysema (>75% predicted KCO) and “moderate” emphysema (40-75% predicted KCO).

### miRNA isolation, hybridization and data extraction

Total RNA was isolated from 30-45 mg of non-tumour lung tissue using Trizol (Invitrogen). DNA was removed using an Ambion DNA-*free*™ kit (Ambion, Foster City, CA, USA) following the manufacturer’s instructions. The resulting total RNA was cleaned using the DNeasy miRNA clean up kit (QIAGEN, Valencia, CA, USA) following manufacturer’s instructions. Purified RNA was quality checked using a Bioanalyzer Agilent Technologies, Santa Clara, CA, USA), based on the presence of miRNAs determined using a small RNA analysis kit and distinct 18S and 28S peaks determined by an mRNA analysis kit. Agilent human miRNA oligo arrays (8×15K array, Part No. G4470A, Agilent Technologies) based on miRBase V9.1 were used. This array contains 15,744 elements representing 470 human miRs and 64 viral miRs. The experiments were performed according to the manufacturer’s instructions. Briefly, 100 ng of total RNA from lung tissue was dephosphorylated using calf intestine alkaline phosphotase to create sticky ends for ligation of the fluorescent dye pCp-Cy3 and T4 RNA ligase (GE Healthcare, Little Chalfont, England). The resulting labeled RNA was purified using Micro-bio spin 6 columns (Bio-Rad, Hercules, CA, USA) to remove unlabeled dye and then hybridized to the Agilent microarray slide for 20 hours at 55°C. The slides were washed to remove any unbound RNA and images were captured using Agilent microarray scanner. Raw features were filtered for spot quality, averaged and extracted using Agilent feature extraction software. The missing values were filled in using the K-nearest neighbor algorithm in Avadis (Strand Life Sciences, Bangalore, India). Study design for microarray experiments satisfied the MIAME guidelines (http://www.mged.org/Workgroups/MIAME/miame_checklist.html). The referred data has been deposited in the Gene Expression Omnibus (GEO) (GSE33338 http://www.ncbi.nlm.nih.gov/gds/?term=GSE33338).

### Candidate miRNA selection and technical validation using qRT-PCR

miRNAs differentially expressed between mild and moderate classes of emphysema lungs were identified by class comparison analysis in BRB-ArrayTools V4.2 (developed by Dr Richard Simon and Amy Peng Lam, freely accessible at http://linus.nci.nih.gov/BRB-ArrayTools.html). A *P* value of <0.01 and false discovery rate (FDR) of <0.05 were used as selection criteria for significance. Two miRNAs were randomly chosen for technical validation by quantitative reverse transcriptase polymerase chain reaction (qRT-PCR) using TaqMan microRNA assays (Applied Biosystems, Foster City, CA, USA). The geometric mean (GeNorm [26]) of two small RNA housekeepers, *U6* and *RNU48*, were used for normalization [26,27].

### Identifying genes modulated by *miR-34c* using *in vitro* techniques

mRNAs targets of altered miRNA expression in commonly used lung cell lines were identified using *in vitro* techniques.

### Cell lines

Commercial lung cell lines BEAS-2B [28] (CRL-9609, a human bronchial epithelial cell line) and HFL1 [29] (CCl-153, a human fetal lung fibroblast cell line) were purchased from ATCC (Virginia, USA). The cell lines were grown as per the supplier’s recommendations. BEAS-2B and HFL1 were cultured in RPMI and DMEM, respectively, supplemented with antibiotics and 10% FCS and incubated in 5% CO_2_.

### Transfection conditions

A microRNA (*miR-34c*) that technically validated and displayed high fold change difference between emphysema classes was chosen for this purpose. Pre-miR™ miRNA precursor molecules (Invitrogen by Life Sciences, Carlsbad, CA) for *miR-34c-5p* were used to increase the expression of the *miR-34c* in HFL1 and BEAS-2B lung cells and the expected increase in expression of *miR-34c* was confirmed using TaqMan microRNAs assays (Invitrogen by Life Sciences, Carlsbad, CA). The -5p isoform of *miR-34c-5p* represents the 5′ arm of the hairpin precursor of the mature miRNA from which the mature sequence has been excised. The probe sequence represented on the microarray was derived from the *miR-34c-5p* sequence. Optimal conditions were tested and selected based on the manufacturer’s instructions. Briefly 50,000 cells were transiently transfected with 20nM pre-miR precursor molecules and NeoFX transfection reagent for 24 hours. The transfection was conducted twice in triplicate each time on two different days two weeks apart. The triplicates were combined for the arrays to provide enough total RNA for the assay.

### mRNA isolation, hybridization and data extraction

Total RNA was extracted and purified from cell lines, BEAS-2B and HFL1, after transfection, using RNeasy Mini kit and RNAse free DNAse kit (QIAGEN, Hilden, Germany). Microarray expression profiling was conducted on the purified RNA using Illumina HT12V3 whole genome gene expression arrays, according to the manufacturer’s instructions. The array contains 48,000 elements representing over 25,000 annotated genes from the RefSeq (Build 36.2) and Unigene databases (Build 199). Element features were extracted using the gene expression module of the BeadStudio V1.1.1 software (GenomeStudio, Illumina, Hayward, CA). Raw features were normalized to the 75th percentile of all elements in GeneSpring GX V9 (Agilent Technologies, CA, USA). Missing values were filled in using the K-nearest neighbor algorithm in Avadis (Strand LifeSciences, Bangalore, India). Differentially expressed genes were identified using class comparison analysis in BRB-ArrayToolV4.2.

### Identification of *miR-34c* predicted targets

The genes differentially expressed between *miR-34c* transfected and non-transfected cells were compared to the predicted targets of miR-34c from the TargetScan and PicTar databases. Candidate target genes whose expression were negatively correlated to that of *miR-34c in vitro* (in cell lines) and *ex vivo* (in lung of TPCH-KCO and Spira *et al.*[19] subjects) were identified. Therefore, identified genes were biologically validated in the original TPCH-KCO [18] samples (used to develop microRNA profiles) by qRT-PCR using pre-designed gene expression assays (Applied Biosystems, Foster City, CA, USA). The raw expression was normalised to the house-keeping gene, GAPDH.

## Results

### Identification of miRNAs associated with emphysema severity using microarrays and confirmation by qRT-PCR

**Figure 1:**
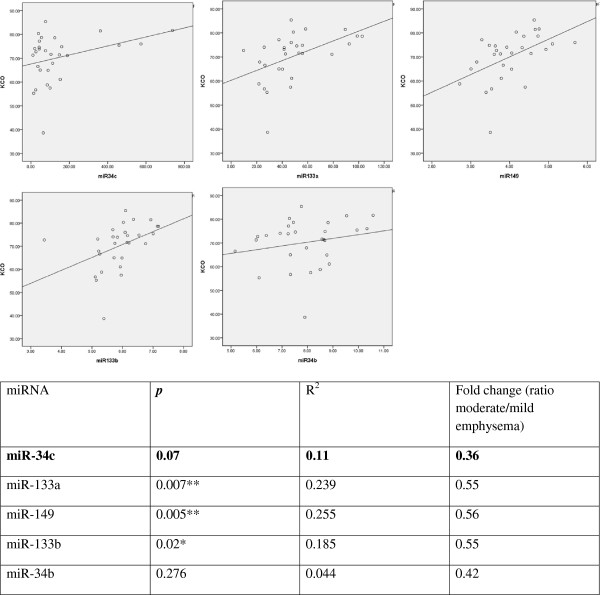
**Correlation plots of the five candidate miRNAs and emphysema status.** The expression of the differentially expressed microRNAs for the 29 patients and their emphysema status (KCO% predicted corrected for hemoglobin) is shown. The correlation of miR34c expression determined by microarrays in lung tissues to its consequent KCO measurements were not significant (*p* > 0.05). However this miRNA was chosen for cell line studies as it portrayed siginificant difference in gene expression between patient groups i.e. moderate vs mild emphysema lungs. MiRNA levels ofthe 29 samples are drawn on the X-axis, KCO% predicted on the left Y-axis and microRNA expression on the right Y-axis. * indicates a correlation of significance *p* < 0.05 and ** indicates a correlation of significance *p* < 0.01.

Agilent human miRNA oligo 8 × 15 K array experiments were conducted on samples of lung from patients with mild (>75% predicted KCO, n = 9) or moderate emphysema (40 to 75% predicted KCO, n = 20). Demographic data are shown in Table 1. After preprocessing of the raw signals, 228 miRNAs were detected in at least 50% of the lung samples. Duplicate array data for these 228 miRNAs were highly correlated (*r*^2^ =0.98, Additional file 1: Figure S1). Class comparison identified five miRNAs (*miR-34c*, *miR-34b*, *miR-149*, *miR-133a* and *miR-133b*) that were significantly differentially expressed between mild and moderate emphysema (p < 0.01, Additional file 1: Figure S2 & Table 2). All five were expressed at lower levels in lung from patients with moderate emphysema, compared with lung from patients with mild emphysema. In this cohort, expression of three of these miRNAs (*miR-149*, *miR-133a* and *miR-133b*) correlated with functional measurement of emphysema severity (KCO, Figure 1). *MiR-34c* expression exhibited the greatest difference between groups with 0.3 fold lower expression in the moderate severity group. qRT-PCR confirmed similar fold differences in expression to microarray results for the two miRNAs, *miR-34c* and *miR-133a* tested (Additional file 1: Figure S3). A flow diagram describing the methods is shown in Additional file 1: Figure S4.

**Table 1:** Demographics of the TPCH-KCO set (n = 29 patients) used in microRNA microarrays

	**TPCH-KCO set**	
	**Mild emphysema**	**Moderate emphysema**
n	9	20
Age (Mean ± SD yrs)	70 ± 4	67 ± 7
Range	63-74	53-78
Male/Female	7/2	14/6
FEV_1_ % predicted (Mean ± SD)	80 ± 17	68 ± 15
Range	52-107	50-97
FEV_1_/VC (Mean ± SD) %	59 ± 5	54 ± 14
Range	50-70	40-70
KCO % predicted (Mean ± SD)	79 ± 3	66 ± 9
Range	75-85	38-74
Pack years (Mean ± SD)	62 ± 34	75 ± 49
Range	28-135	24-240
Site of tissue collection	1-LLL	1-LL
	1-RLL	5-LUL
	1-LL	7-RLL
	1-RL	5-RUL
	5-RUL	2-RML
GOLD Stage I Mild (FEV_1_ ≥80% predicted)	4	3
GOLD Stage II Moderate (50 < FEV_1_ < 80% predicted)	5	17

*List of abbreviations*, *FEV1* forced expired volume in one second, *VC* vital capacity, *KCO* transfer coefficient of carbon monoxide, *SD* standard deviation, *LLL* left lower lobe, *RLL* right lower lobe, *LUL* left upper lobe, *RUL* right upper lobe, *RML* right middle lobe, *LL* left lung, *RL* right lung.

**Table 2:** Demographics of miRNAs significantly downregulated in the moderate emphysema patients compared with mild emphysema patients

**Unique id**	**p-value**	**False discovery rate (FDR)**	**Geometric mean of class 1 (moderate)**	**Geometric mean of class 2 (mild)**	**Fold-change**
hsa-miR-149	0.001	0.019	13.60	24.24	0.56
hsa-miR-133a	0.002	0.019	35.66	64.71	0.55
hsa-miR-133b	0.003	0.019	49.72	90.43	0.55
hsa-miR-34c	0.006	0.034	57.53	158.14	0.36
hsa-miR-34b	0.008	0.034	185.41	437.64	0.42

### Effect of *miR-34c-5p* over-expression on its predicted mRNA targets in respiratory cells *in vitro* using gene expression microarrays

To identify mRNA targets regulated by *miR-34c* in lung cells, we transfected *miR-34c-5p* precursor into BEAS-2B and HFL1 cells, and demonstrated the expected 95-99% increased expression of *miR-34c* by qRT-PCR (data not shown). Illumina HumanHT12 V3 transcriptome microarrays were then used to examine the expression of mRNAs in *miR-34c*-transfected cells, relative to cells transfected with scrambled sequence controls. Class comparison analysis in BRB-ArrayTools V4.2 identified 2,152 elements (48 expected by chance) affected by over-expression of *miR-34c* (*p* < 0.001) in BEAS-2B and HFL1 cell lines, the vast majority of which (90%) were down-regulated by elevated *miR-34c* levels. Among the 2,152 genes, fifty were predicted as miR-34c targets by the databases Pictar and TargetScan-and they were significantly down-regulated (>1.8 fold down-regulation) in the transfectants. We next examined *ex vivo* expression patterns of the genes found to be *miR-34c* responsive *in vitro.* Of the fifty genes, thirty-three were represented in the filtered TPCH-KCO dataset [18] and thirty-two in the dataset of Spira *et al.*[19].

**Figure 2:**
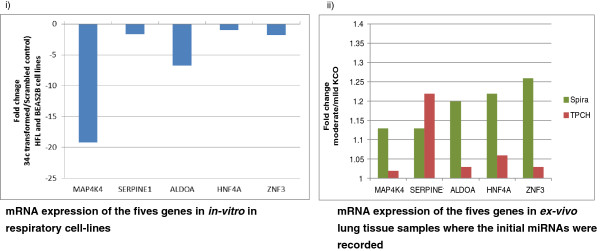
**Histogram of in vitro (i) and *ex vivo *(ii) expression of genes predicted and modulated by *miR-34c *over-expression in BEAS-2B and HFL1 cell lines.** Ratio of moderate to mild gene expression of emphysema patients are shown on the Y-axis and the genes on the X-axis.

**Figure 3:**
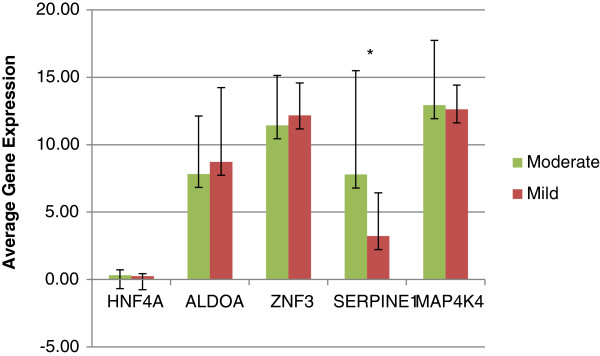
**Histogram of qRT-PCR measurements of miR-34c regulated targets in TPCH-KCO dataset.** Genes differentially expressed between transformed and scrambled sequence controls in respiratory cell lines were independently tested in TPCH-KCO dataset using qRT-PCR. This step minimizes false positives and ensures that authentic genes are discovered. Average gene expression in mild and moderate emphysema lungs are represented in the Y-axis and genes on the X-axis. SERPINE1 was significantly upregulated in emphysema lungs. HNF4 was also upregulated but did not attain significance.

In both of these datasets, five genes (*MAP4K4*, *SERPINE1*, *ALDOA*, *HNF4A* and *ZNF3*) were expressed at higher levels in lung from patients with moderate or severe emphysema compared with lung from normal subjects or subjects with mild emphysema, whereas expression of *miR-34c* was reciprocal (Figure 2), consistent with the possibility that these five genes may be regulated by *miR-34c* in emphysema. In the 29 *ex vivo* lung samples used for miRNA microarray profiling, qRT-PCR confirmed that expression of both *SERPINE1* (fold-change >2.2) and *HNF4A* (fold-change >1.3) had generally higher expression in association with lower miR-34c expression; however only *SERPINE1* (*p* = 0.05) was significantly differentially expressed between mild and moderate emphysema (Figure 3) in this cohort, and it was correspondingly upregulated in more severe disease in both TPCH-KCO (fold change >1.2, *p* < 0.05) and Spira (fold change >1.13) datasets (Figure 2).

## Discussion

MiRNAs regulate the expression of genes involved in biological processes relevant to the progression of chronic lung disease, including cellular stress, cell differentiation and apoptosis [30-32]. Here, we aimed to identify miRNAs (and their target mRNAs) associated with emphysema severity in COPD patients. Five miRNAs were identified to be significantly down-regulated in lung from patients with moderate emphysema, compared with lung of mild emphysema patients. In addition, a negative correlation was noted in the expression of these miRNAs and their putative target mRNAs *ex vivo.* Furthermore, increasing the expression of *miR-34c* (the miRNA with the largest fold change between the mild and moderate emphysema patient groups) in respiratory cells resulted in decreased expression of predicted mRNA targets *in vitro*, providing additional functional expression data. Our data suggest modulation of SERPINE1 by *miR-34c in vitro* and *ex vivo*. Dysregulation of *SERPINE1* by *miR-34c* could therefore be a potential mechanism involved in emphysema severity and progression. To our knowledge, this is the first study to compare miRNA profiles in the lungs of COPD patients based on emphysema severity.

A major strength of this study was the ability to correlate miRNA-mediated mRNA regulation in the same lung samples. It is known that miRNAs mediate gene silencing through translational inhibition and mRNA degradation. In addition, target mRNA is degraded in more than one third of genes that display translational repression [33]; hence we have focused on studying changes in mRNA expression as a consequence of altered miRNA expression. MiRNAs that are down-regulated in a certain disease state (e.g. moderate emphysema, compared to mild emphysema) would be expected to upregulate expression of their predicted mRNAs, in an orchestrated process of epigenetic regulation. This relationship of miRNA and mRNA expression has been demonstrated previously in airways from COPD patients [25] but not in peripheral lung tissue, which we used in this current study. Of the miRNAs identified as differentially expressed in lung between moderate vs mild emphysema patients in this study, *miR-34c* is particularly relevant to human lung disease, as its expression is increased during normal lung development [34]. We found decreased expression of *miR-34c* in lungs of patients with moderate emphysema, compared to mild emphysema. Van Pottelberge *et al.* also found greater than three-fold downregulation of *miR-34c* in sputum from smokers with COPD, compared to those without COPD, and a direct correlation between *miR-34c* expression and percent predicted FEV_1_[24]. Their findings support our study, since there was down-regulation of *miR-34c* expression in their COPD patients, compared to controls, and also down-regulation in our moderate emphysema patients, compared to mild emphysema, indicating a similar continuum of expression with increasing disease severity. In the setting of reduced *miR-34c* expression in moderate emphysema lung (vs mild), we were able to confirm increased gene expression of two of its predicted mRNA targets, *HNF4* and *SERPINE1*. HNF4A (hepatocyte nuclear factor 4A) is a transcription factor that regulates the expression of cytokines involved in inflammation, as demonstrated in other organs (e.g. kidney [35]); its role in the lung is yet to be determined. *SERPINE1* (Serpin Peptidase Inhibitor, Clade E (Nexin, Plasminogen Activator Inhibitor Type 1), Member 1), also known as plasminogen activator inhibitor 1 (*PAI-1)*, is a protease inhibitor, and inhibitor of fibrinolysis. *PAI-1* knockout mice demonstrate emphysema-like changes in the lung [36], so our findings of increased *SERPINE1* expression in moderate emphysema (vs mild emphysema) may be unexpected, given the role of protease-antiprotease imbalance in the pathogenesis of emphysema. However, SERPINE1 is only one of a number of antiproteases, and has other roles in the lung that could be relevant to the progression of emphysema. For example, *SERPINE1* has been shown to be upregulated in the lung or lung cells during hypoxia [37], exposure to lipopolysaccharide or cigarette smoke extract [38], or oxidative stress via NF-κB [39]. Importantly, elevated levels of PAI-1 in sputum have been observed in two separate studies of patients with COPD (compared to controls) [39,40]. Furthermore, sputum PAI-1 levels were higher in more severe COPD (compared to milder COPD), which would be in keeping with our findings of higher *SERPINE1* expression in more severe emphysema [40]. Other candidate emphysema severity miRNAs identified in this study – *miR-34b*, *miR-133a/b* and *miR-149* – have been previously implicated in the pathogenesis of lung diseases. For example, *miR-34b* expression was decreased in induced sputum from COPD smokers compared to non-COPD smokers [24]. Down-regulation of *miR-133a* is associated with increased bronchial smooth muscle contraction in patients with asthma [41]. *miR-133b* negatively regulates the expression of transcription factor, *Pitx3*[42], which activates the dopamine receptor (DRD1), a mediator of nicotine addiction in smokers [43]. Overall, emerging evidence of the biological roles of these miRNAs supports the plausibility of their involvement in lung diseases.

A number of potential limitations of this study should be addressed. (i) A physiological measure of emphysema (gas transfer) was used to stratify patients, as in our previous study [18], rather than pathological examination of lung tissue. The reason for this was that these lung tissue samples were not inflated at harvesting, making morphological assessment of emphysema severity difficult. However, gas transfer is routinely used clinically as a measure of emphysema severity in COPD patients. (ii) Although a relatively small number of samples was used (from 29 patients), the FDRs of the 5 miRNAs were all <0.05; furthermore, the two miRNAs tested (*miR-34c* and *miR-133a*) were technically validated independently using qRT-PCR. (iii) No overlap was found between the *miR-34c* targets that were enriched in *ex vivo* lung samples and *in vitro* cell lines. It should be noted that the *ex vivo* study used Operon microarrays which represented only 14,000 genes. Furthermore, our *in vitro* study used commercial cell lines, and all of our COPD lung samples were from patients who also had lung cancer. Despite these potential limitations, we found that the expression of five candidate genes *in vitro* correlated with *ex vivo* profiles in both in-house [18] and external datasets (Spira *et al.*[19]).

## Conclusion

In summary, miRNA expression is associated with increasing emphysema severity in COPD patients. Increasing *miR-34c* expression *in vitro* in respiratory cell lines decreased the expression of its predicted targets, consistent with miRNA-mediated regulation of mRNA. Additionally, we have shown by qRT-PCR that *SERPINE1* expression is increased in lung from patients with increasing severity of emphysema, in whom *miR-34c* expression is low. Future studies should aim to directly demonstrate *miR-34c*-mediated *SERPINE1* regulation in emphysema and evaluate the pathogenetic role of other miRNAs, with the goal of identifying potentially important targets for more effective treatment of COPD.

### Availability of supporting data

The data sets supporting the results of this article are available in the Gene Expression Omnibus (GEO), identified as GSE33338 (http://www.ncbi.nlm.nih.gov/gds/?term=GSE33338).

## Competing interests

The authors declare that they have no competing interests.

## Authors’ contributions

SS carried out all the experiments, analysis and drafted the manuscript. MD participated in the initial planning of experiments and analysis of results. MT helped with cell line maintenance and gene expression arrays. CW helped with gene expression arrays. BC and ED collected and graded all lung tissue specimens used in the study. RB, NK, KF and IY participated in the planning of the study design, experiments and analysis. IY helped with the drafting of the manuscript. All authors read and approved the final manuscript.

## Supplementary Material

Additional file 1: Figure S1 Graphical comparison of technical replicates used in the miRNA microarrays on Agilent Human miRNA profiler. Two lung samples were randomly chosen to be repeated on different array on different days to evaluate the reproducibility of the arrays. Raw signal intensity of all 550 miRNAs is shown for the replicate samples. **Figure S2.** Dendrogram of the i) 228 filtered miRNAs used in the class comparison analysis and ii) the five miRNAs significantly differentially expressed between mild and moderate emphysema (*p*<0.01). The red and green colours indicate miRNAs over and under expressed in the mild compared with moderate emphysema patients, respectively. The yellow and blue bars indicate mild and moderate emphysema samples, respectively. **Figure S3.** Technical validation of microRNA expression for two microRNAs. A) Histogram of microRNA expression measured by qRT-PCR and microarray. The expression is shown as the ratio of moderate to mild emphysema patients on the Y-axis for both methods. B) Correlation plots of microRNA expression measured by qRT-PCR versus microarrays. **Figure S4.** Analytical flow diagram describing the miRNA microarray data analysis, miRNA identification, miRNA-mRNA target correlation and in vitro validation. Click here for file
