# Supplementary material for: MicroRNA-34c is associated with emphysema severity and modulates SERPINE1 expression
Source: BMC Genomics. 2014 Jan 30;15:88. doi: 10.1186/1471-2164-15-88 (PMC3922660; doi:10.1186/1471-2164-15-88)
Supplement: Additional file 1: Figure S1 — Graphical comparison of technical replicates used in the miRNA microarrays on Agilent Human miRNA profiler. Two lung samples were randomly chosen to be repeated on different array on different days to evaluate the reproducibility of the arrays. Raw signal intensity of all 550 miRNAs is shown for the replicate samples. Figure S2. Dendrogram of the i) 228 filtered miRNAs used in the class comparison analysis and ii) the five miRNAs significantly differentially expressed between mild and moderate emphysema (p<0.01). The red and green colours indicate miRNAs over and under expressed in the mild compared with moderate emphysema patients, respectively. The yellow and blue bars indicate mild and moderate emphysema samples, respectively. Figure S3. Technical validation of microRNA expression for two microRNAs. A) Histogram of microRNA expression measured by qRT-PCR and microarray. The expression is shown as the ratio of moderate to mild emphysema patients on the Y-axis for both methods. B) Correlation plots of microRNA expression measured by qRT-PCR versus microarrays. Figure S4. Analytical flow diagram describing the miRNA microarray data analysis, miRNA identification, miRNA-mRNA target correlation and in vitro validation. [file 1471-2164-15-88-S1.doc]

**List of Supplementary Figures**

**Figure E1:** Graphical comparison of technical replicates used in the miRNA microarrays on Agilent Human miRNA profiler. Two lung samples were randomly chosen to be repeated on different array on different days to evaluate the reproducibility of the arrays. Raw signal intensity of all 550 miRNAs is shown for the replicate samples.

**Figure E2:** Dendrogram of the i) 228 filtered miRNAs used in the class comparison analysis and ii) the five miRNAs significantly differentially expressed between mild and moderate emphysema (*p*<0.01). The red and green colours indicate miRNAs over and under expressed in the mild compared with moderate emphysema patients, respectively. The yellow and blue bars indicate mild and moderate emphysema samples, respectively.

**Figure E3:** Technical validation of microRNA expression for two microRNAs. A) Histogram of microRNA expression measured by qRT-PCR and microarray. The expression is shown as the ratio of moderate to mild emphysema patients on the Y-axis for both methods. B) Correlation plots of microRNA expression measured by qRT-PCR versus microarrays.

**Figure E4:** Analytical flow diagram describing the miRNA microarray data analysis, miRNA identification, miRNA-mRNA target correlation and in vitro validation.


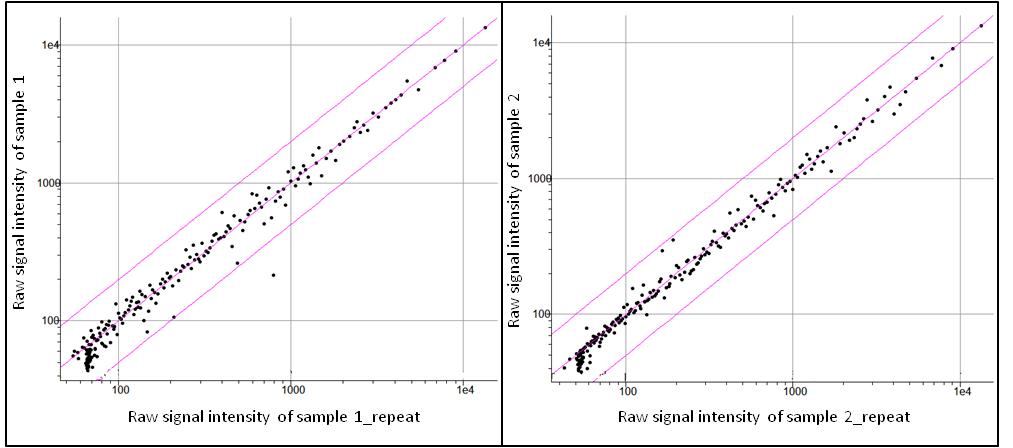
**Figure E1**


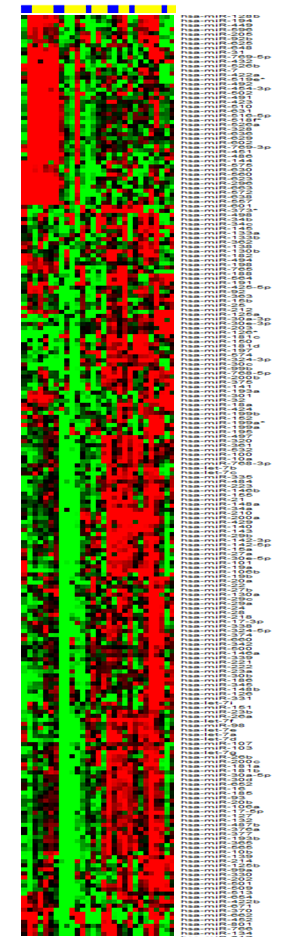

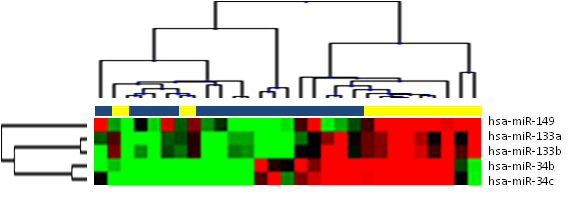
 **Figure E2**

All 228 miRNAs

5 differentially expressed miRNAs

**i)**

**ii)**

**Figure E3**

A


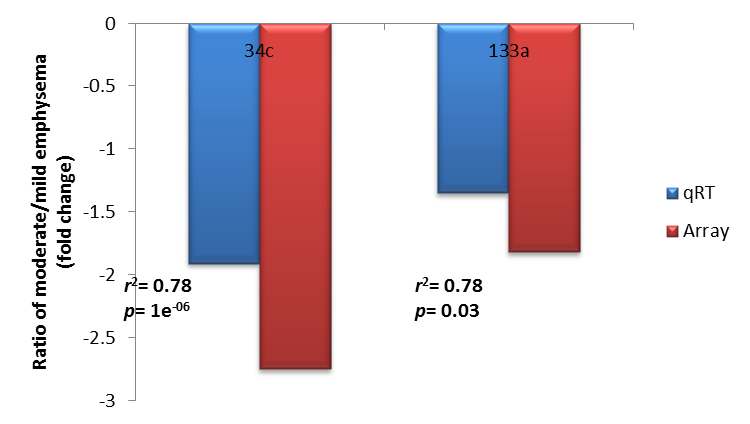


hsa-miR-34c


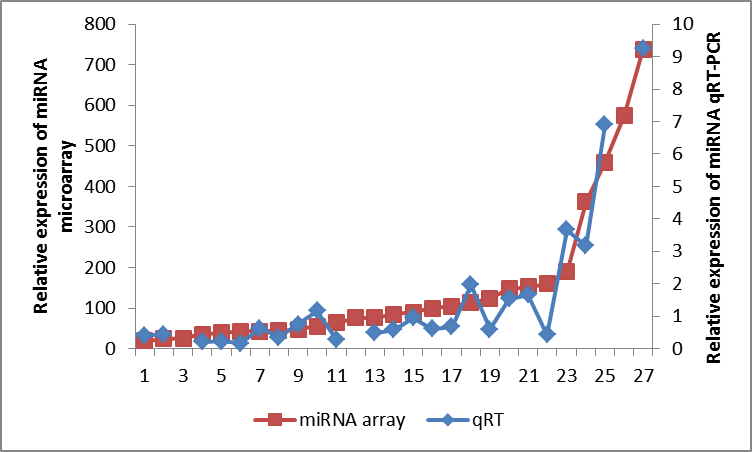
B

*r*^2^= 0.78

*p*= 1 e-06


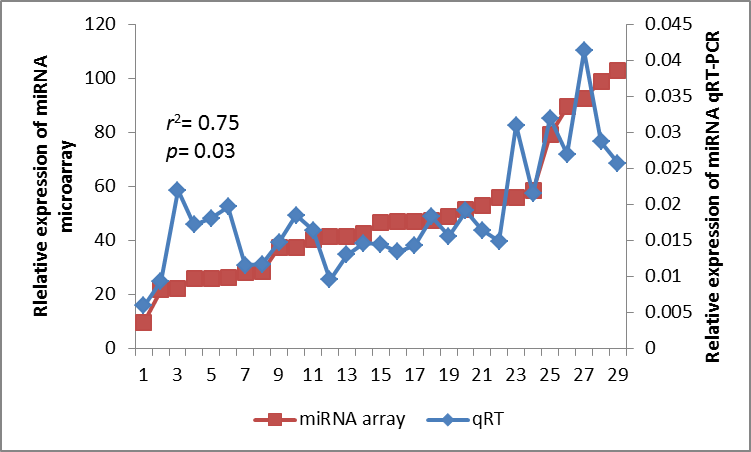


hsa-miR-34c

hsa-miR-133a

**Figure E4**

Transcriptomics miRNAomics

Mild vs moderate emphysema

Mild= KCO>75% predicted, Moderate=KCO <75% predicted

TPCH dataset

Operon V2.1 microarray (~14,000 genes) Agilent miRNA microarray G4470 V1.0 (~500 miRNAs)

Filtered genelist (n=6420) *DE microRNAs p<0.01 (n=5)

*miR-34c, miR-34b, miR-133a, miR-133b, miR-149*

*miR-34c* displayed highest fold change (moderate/mild)

*Ex Vivo*

*In Vitro*

Increasing *miR-34c* and its mRNA target regulation in cells lines

HFL 1 cells BEAS-2B cells

Scrambled Control vs miR-34c

Illumina Human HT12 V3.0 Arrays

Genes *DE (p<0.001) and down-regulated in miR34c tranfectant

(n=2152)

TPCH-KCO & Spira *et al* dataset Overlap with PicTar and TargetScan predicted targets

N=5 down-regulated genes identified with miR-34c transfection

(*MAP4K4, ZNF3, SERPINE1, HNF4A, ALDOA*)

qRT-PCR validation of all 5 genes

**SERPINE1 and HNF4 were technically validated

*DE: Differentially expressed; ** *p*value=0.05

**indicates a significant correlation at p<0.05 and ** indicates a significant correlation at p<0.1*
